# Electrocardiogram abnormalities and prognosis in COVID-19

**DOI:** 10.3389/fcvm.2022.993479

**Published:** 2022-10-05

**Authors:** Gabriel Chevrot, Marie Hauguel-Moreau, Marion Pépin, Antoine Vieillard-Baron, Anne-Sophie Lot, Mounir Ouadahi, Hélène Hergault, Vincent Aïdan, Ségolène Greffe, Adrien Costantini, Olivier Dubourg, Sébastien Beaune, Nicolas Mansencal

**Affiliations:** ^1^Department of Cardiology, Ambroise Paré Hospital, Assistance Publique-Hôpitaux de Paris (AP-HP), Centre de référence des cardiomyopathies et des troubles du rythme cardiaque héréditaires ou rares, Université de Versailles-Saint Quentin (UVSQ), Boulogne-Billancourt, France; ^2^INSERM U-1018, CESP, Epidémiologie clinique, UVSQ, Université de Paris Saclay, Villejuif, France; ^3^Department of Geriatrics, Ambroise Paré Hospital, AP-HP, UVSQ, Boulogne-Billancourt, France; ^4^Intensive Care Unit, Ambroise Paré Hospital, AP-HP, UVSQ, Boulogne-Billancourt, France; ^5^FHU SEPSIS IFrancenserm UMR 1144, Université Paris Centre, Paris, France; ^6^Department of Medical Information, Ambroise Paré Hospital, APHP, Boulogne-Billancourt, France; ^7^Department of Internal Medicine, Ambroise Paré University Hospital, AP-HP, UVSQ, Boulogne-Billancourt, France; ^8^Pneumology Department, Ambroise Paré Hospital, AP-HP, UVSQ, Boulogne-Billancourt, France; ^9^Department of Emergency Medicine, Ambroise Paré Hospital, AP-HP, UVSQ, Boulogne-Billancourt, France

**Keywords:** ECG, COVID-19, prognosis, SARS-CoV-2, repolarization

## Abstract

**Background:**

COVID-19 is a major pandemic with potential cardiovascular complications. Few studies have focused on electrocardiogram (ECG) modifications in COVID-19 patients.

**Method and results:**

We reviewed from our database all patients referred to our hospital for COVID-19 between January 1st, 2020, and December 31st, 2020: 669 patients were included and 98 patients died from COVID-19 (14.6%). We systematically analyzed ECG at admission and during hospitalization if available. ECG was abnormal at admission in 478 patients (71.4%) and was more frequently abnormal in patients who did not survive (88.8 vs. 68.5%, *p* < 0.001). The most common ECG abnormalities associated with death were left anterior fascicular block (39.8 vs. 20.0% among alive patients, *p* < 0.001), left and right bundle branch blocks (*p* = 0.002 and *p* = 0.02, respectively), S1Q3 pattern (14.3 vs. 6.0%, *p* = 0.006). In multivariate analysis, at admission, the presence of left bundle branch block remained statistically related to death [OR = 3.82, 95% confidence interval (CI): 1.52–9.28, *p* < 0.01], as well as S1Q3 pattern (OR = 3.17, 95% CI: 1.38–7.03, *p* < 0.01) and repolarization abnormalities (OR = 2.41, 95% CI: 1.40–4.14, *p* < 0.01).

On ECG performed during hospitalization, the occurrence of new repolarization abnormality was significantly related to death (OR = 2.72, 95% CI: 1.14–6.54, *p* = 0.02), as well as a new S1Q3 pattern (OR = 13.23, 95% CI: 1.49–286.56, *p* = 0.03) and new supraventricular arrhythmia (OR = 3.8, 95% CI: 1.11–13.35, *p* = 0.03).

**Conclusion:**

The presence of abnormal ECG during COVID-19 is frequent. Physicians should be aware of the usefulness of ECG for risk stratification during COVID-19.

## Introduction

Coronavirus Disease 2019 (COVID-19) is a main pandemic infection that has hit the world with multiple waves (1). Its evolution through the years ahead remains uncertain (2), because of the emergence of new variants (3, 4), vaccination campaigns and innovative treatments (5).

Previous cardiovascular comorbidities seem to worsen the prognosis of the infection (6), but COVID-19 may cause several cardiovascular complications *via* different mechanisms (7). Systemic inflammation can destabilize vascular plaque, while viral illness increases cytokine activity, increasing cardiac demand, like influenza (8). Severe acute respiratory syndrome coronavirus 2 (SARS-CoV-2) may also cause direct damage to the heart utilizing ACE2 receptors located within cardiac tissue (9). This infection is thereby associated with venous thromboembolic events (10–14), myocarditis (7, 15, 16), arrhythmias (14) and increased risk of acute myocardial infarction (17–20) and possible coronary tropism of the virus in thrombi (21, 22). All these complications may induce electrocardiographic abnormalities. Electrocardiogram (ECG) is a simple and broadly available exam which can be rapidly performed without exposing a large number of staff to the virus. Systematic standard ECG may be a useful screening tool for cardiovascular complications in patients presenting with COVID-19.

There are few published studies of ECG modifications related to COVID-19 (23–28). A retrospective cohort study of 756 patients comparing ECG abnormalities showed that both left- and right-sided heart disease in patients with COVID-19 have higher odds of death (26), but the confounding factors are not well-described.

A narrative review shows that up to 90% of critically ill patients have at least one ECG abnormality, including supraventricular tachycardia or ST modification, mainly related to cytokine storm, hypoxic injury, electrolyte abnormalities, plaque rupture, coronary spasm, microthrombi, or direct endothelial or myocardial injury (27). The objective of this study was to describe ECG abnormalities during COVID-19 and their impact on prognosis.

## Methods

We reviewed from our database all patients referred to our hospital for COVID-19 between January 1st, 2020 and December 31st, 2020. All the cases of COVID-19 were proved by SARS-CoV-2 RT-PCR on nasopharyngeal swabs. We included all hospitalized patients [including in intensive care unit (ICU)] with an available ECG. Patients with ventricular pacing were excluded.

Baseline characteristics of the population were systematically recorded: demographic characteristics (sex, body mass index, age), habits (smoking status), medical background (diabetes, hypertension, dyslipidemia, ischemic heart disease, history of familial cardiovascular disease, heart failure, history of supraventricular arrhythmia, history of venous thromboembolic disease, known pulmonary hypertension, peripheral arterial disease, history of stroke, chronic obstructive pulmonary disease or asthma, chronic lung failure, i.e., requiring home oxygen therapy, chronic kidney disease, defined as glomerular filtration rate < 60 ml/kg/m^2^, active cancer or immunosuppression), initial presentation (including time from symptoms onset), and hospitalization duration. We assessed several biomarkers at admission: leukocytes, C-reactive protein, hemoglobin, D-dimers, potassium, creatinine and troponin. We also recorded the main drugs that were administered to the patient (including heparin, oral anticoagulant drugs, antibiotics, immunotherapy, catecholamine, steroids) and major events that occurred during hospitalization, including pulmonary embolism, intensive care admission, extracorporeal life support, type of ventilation (non-invasive oxygen therapy, high-flow oxygen therapy, continuous positive airway pressure, mechanical ventilation).

Electrocardiograms at admission were systematically analyzed. We also assessed potential occurrence of new ECG abnormalities in patients with ECG performed during hospitalization. In case of multiple ECGs, we considered the first ECG done during hospitalization. The ECG interpretation was performed by two independent physicians blinded to clinical status and outcome. Any disagreement in interpretation between readers was resolved by consensus. The following ECG parameters were systematically assessed: (1) heart rate, (2) sinus rhythm status, (3) atrial and ventricular arrhythmias, (4) PR duration (in milliseconds), (5) atrioventricular block, (6) low voltage (defined as low amplitude of QRS complexes of < 10 mm in precordial leads, or < 5 mm in frontal leads), (7) Q wave, (8) S1Q3 pattern, (9) QRS duration and QRS abnormalities including bundle branch block and QRS axis, (10) repolarization abnormalities (negative T wave and/or unspecific repolarization abnormalities, ST elevation), (11) QT corrected measured with the Bazett formula, and (12) atrial or ventricular premature ventricular complexes. Prolonged QTc was defined as QTc > 460 ms.

The primary outcome was death occurring during hospitalization.

Statistical analysis was performed using MedCalc software (Ostend, Belgium) or with R Development Core Team (29) (R: A language and environment for statistical computing. R Foundation for Statistical Computing, Vienna, Austria). Continuous variables with normal distribution are presented as mean ± SD and compared using Student's *t*-test. Continuous variables with non-normal distribution are presented as median [interquartile range (IQR)] and are compared using the Mann-Whitney U test. Qualitative variables are presented as variable (percentage) and compared using the Chi squared test or Fisher's exact test. Multivariate logistic regression models were used to assess the association between ECG abnormalities and death. We included in each logistic regression model baseline clinical characteristics and ECG abnormalities that were clinically relevant and possibly associated with death in univariate analysis (*p* < 0.20) using forward stepwise selection. The results are interpreted in terms of adjusted odds ratios with their associated 95% confidence interval. We performed a second multivariate analysis to identify variables independently associated with death among patients who had a second ECG during hospitalization. Sensitivity analyses were performed and did not modify the outcomes. Statistical tests were considered significant for a *P*-value < 0.05. This study was approved by the University Paris Saclay Institutional Review Board (CER-Paris-Saclay-2021-102).

## Results

**Figure 1:**
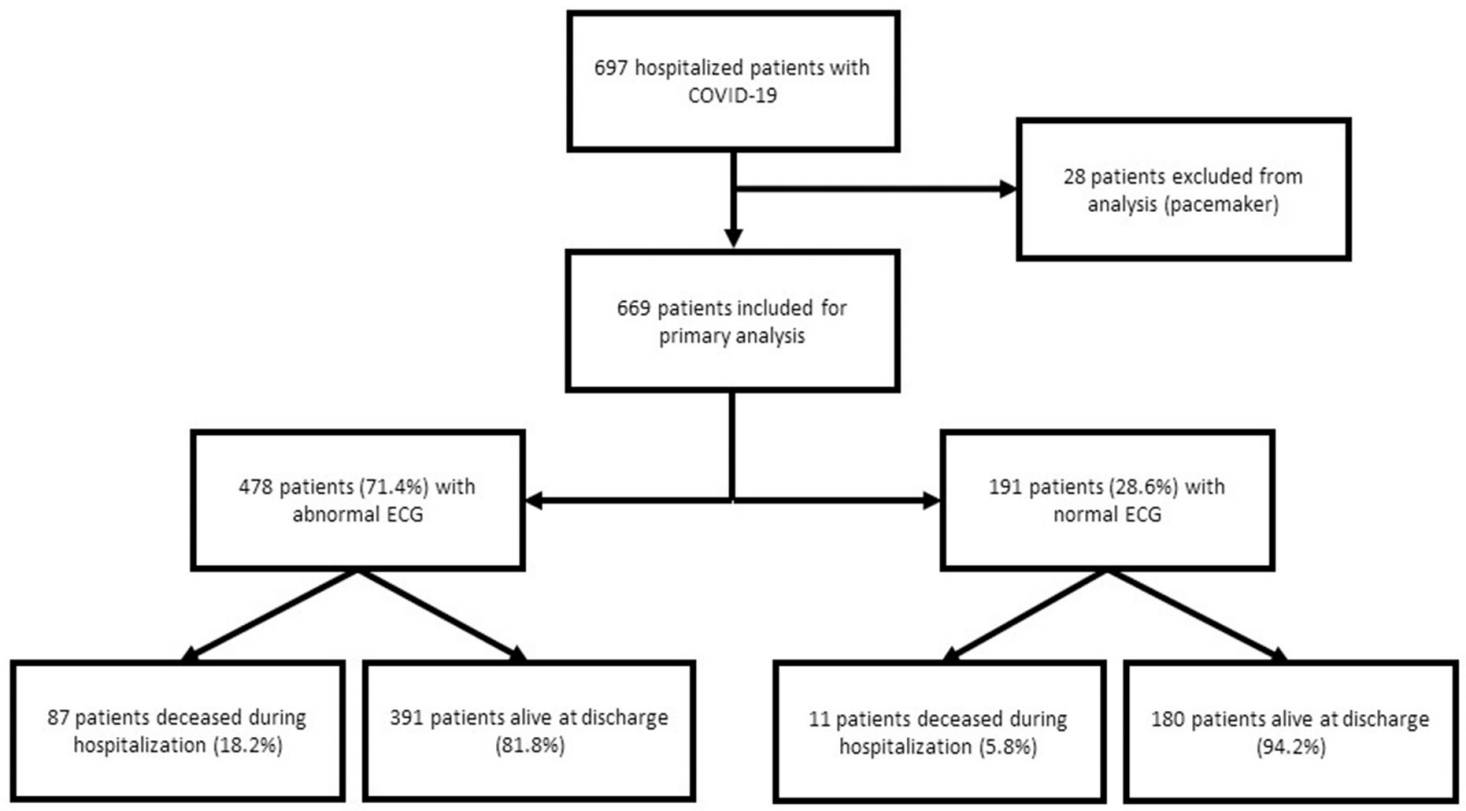
Flowchart of the study.

We included 697 patients hospitalized with confirmed COVID-19. Of these patients, 28 were excluded because of pacing (Figure 1), and 669 patients were included in the primary analysis. Baseline characteristics of the global population and according to vital status are presented in Table 1. One hundred and seventy-four patients (26.0 %) were admitted in ICU. Ninety-eight patients (14.6%) died from COVID-19 during hospitalization. Mean age was 69.1 ± 17.2 years and was significantly older in deceased patients (80.9 ± 11.1 years vs. 67.1 ± 17.3 years, *p* < 0.001). Deceased patients more frequently had hypertension (67.3 vs. 45%, *p* < 0.001) and dyspnea (73.5 vs. 52.7%, *p* < 0.001). Mean leukocyte count was 8.2 ± 4.3 G/L and was statistically higher in the non-survival group (*p* = 0.001), as were CRP level (*p* < 0.001), D-dimers (*p* = 0.01), serum creatinine (*p* < 0.001), troponin (*p* = 0.03) and NT pro BNP (*p* < 0.001). The median time from symptoms onset to admission was 7 days, without any statistical difference between the two groups (*p* = 0.057). Pulmonary embolism was diagnosed in 37 patients (5.5%), heart failure in 45 patients (6.7%), acute coronary syndrome in 10 patients (1.5%) and pericarditis in 2 patients (0.3%). No myocarditis was observed.

**Table 1:** Baseline characteristics of the study population presenting with COVID-19.

	**Alive,** ***n* = 571 (85.4%)**	**Dead,** ***n* = 98 (14.6%)**	**P value**
**Clinical characteristics**			
Male	330 (57.8%)	62 (63.3%)	0.365
Body mass index, kg/m^2^	26 ± 5.6	25.9 ± 6.8	0.845
Age, years	67.1 ± 17.3	80.9 ± 11.1	< 0.001
Smoker	44 (7.7%)	7 (7.1%)	0.990
Diabetes mellitus	105 (18.4%)	26 (26.5%)	0.082
Hypertension	257 (45%)	66 (67.3%)	< 0.001
Dyslipidemia	152 (26.6%)	37 (37.8%)	0.032
History of familial cardiovascular disease	6 (1.1%)	1 (1%)	0.610
Coronary artery disease	61 (10.7%)	20 (20.4%)	0.011
Heart failure	16 (2.8%)	11 (11.2%)	< 0.001
Supraventricular arrhythmia	63 (11%)	20 (20.4%)	0.015
History of pulmonary embolism or thrombosis	31 (5.4%)	9 (9.2%)	0.223
Pulmonary hypertension	1 (0.2%)	0 (0%)	0.317
Peripheral arterial disease	21 (3.7%)	12 (12.2%)	< 0.001
History of stroke	31 (5.4%)	14 (14.3%)	0.116
Chronic obstructive pulmonary disease or asthma	77 (13.5%)	14 (14.3%)	0.957
Chronic lung failure	24 (4.2%)	3 (3.1%)	0.800
Active cancer	24 (4.2%)	17 (17.3%)	< 0.001
Chronic kidney disease	35 (6.1%)	14 (14.3%)	0.008
**Initial symptoms**			
Cough	275 (48.2%)	45 (45.9%)	0.763
Dyspnea	301 (52.7%)	72 (73.5%)	< 0.001
Chest pain	60 (10.5%)	3 (3.1%)	0.032
Syncope or faintness	92 (16.1%)	14 (14.3%)	0.758
Asthenia	238 (41.7%)	30 (30.6%)	0.051
Anosmia or ageusia	54 (9.5%)	4 (4.1%)	0.121
Gastrointestinal symptoms	174 (30.5%)	17 (17.3%)	0.011
Time from symptom onset to hospitalization	7 [4–10]	6 [3–10]	0.057
Heart rate per min	91 ± 20	89 ± 21	0.343
Systolic blood pressure, mmHg	137 ± 24	133 ± 23	0.131
Diastolic blood pressure, mmHg	76 ± 14	73 ± 18	0.116
Temperature, °C	37.5 ± 1.0	37.6 ± 1.2	0.660
Oxygen saturation, %	95 [92–97]	93 [87–95]	< 0.001
Respiratory rate, per min	25 [20–30]	30 [24–36]	< 0.001
**Laboratory data**			
Leukocytes (G/L)	8.0 ± 4.1	9.6 ± 5.2	0.001
CRP (mg/L)	77 [33–151]	127 [66–205]	< 0.001
Hemoglobin (g/dL)	13.3 ± 1.9	12.9 ± 2.2	0.022
D-dimers (ng/mL)	792 [702–1,320]	1,127 [806–1,958]	0.011
Serum potassium (mmol/L)	4.2 ± 0.5	4.2 ± 0.5	0.656
Serum creatinine (μmol/L)	75 [73–77]	96 [90–106]	< 0.001
Cardiac troponin I at admission (μg/L)	0.043 [0.022–0.109]	0.083 [0.034–0.455]	0.028
NT pro BNP (ng/L)	526 [150–1,895]	1,810 [632–6,433]	< 0.001
**Management**			
Intensive care unit admission	132 (23.1)	42 (42.9)	< 0.001
Oxygen therapy	409 (71.6)	95 (96.9)	< 0.001
High-flow nasal oxygen therapy	77 (13.5)	24 (24.5)	0.008
Continuous positive airway pressure	7 (1.2)	4 (4.1)	0.104
Mechanical ventilation	30 (5.3)	25 (25.5)	< 0.001
Hospitalization duration	9 [5–17]	10 [5–17]	0.504

All results are presented as mean (%), mean ± SD, or median (25th to 75th percentiles).

### ECG characteristics at admission

The ECG characteristics at admission are presented in Table 2. All patients had an ECG at admission, and 169 patients (25.3%) had another ECG during hospitalization. An abnormal ECG at admission was observed in 478 patients (71.4%) and was more frequently present in patients who did not survive (88.8 vs. 68.5%, *p* < 0.001). An abnormal ECG was also more frequently present in patients admitted in ICU than in non-ICU patients (79.3 vs. 68.7%, *p* < 0.01). The most common abnormality associated with death was left anterior fascicular block (39.8 vs. 20.0%, *p* < 0.001). Left and right bundle branch blocks were also statistically associated with death (10.2 vs. 3.0%, *p* = 0.002 and 12.2 vs. 5.3%, *p* = 0.02, respectively), as were S1Q3 pattern (14.3 vs. 6.0%, *p* = 0.006) and non-specific repolarization abnormalities (30.6 vs. 14.4%, *p* < 0.001). Mean corrected QT interval was 439 ± 31 ms (446 ± 36 ms among non-survival patients vs. 435 ± 29 ms among patients who survived, *p* = 0.006).

**Table 2:** ECG characteristics at admission in patients presenting with COVID-19.

	**Alive,** ***n* = 571 (85.4%)**	**Dead,** ***n* = 98 (14.6%)**	***P*-value**
Abnormal ECG	391 (68.5%)	87 (88.8%)	< 0.001
Supraventricular arrhythmia	52 (9.1%)	14 (14.3%)	0.162
PR duration, ms	157 ± 29	168 ± 41	0.004
Atrioventricular block, first degree	42 (8.1%)	16 (19.3%)	0.003
Atrioventricular block, second degree	0 (0%)	0 (0%)	–
Atrioventricular block, third degree	0 (0%)	0 (0%)	–
Low voltage	51 (8.9%)	9 (9.2%)	0.912
Q wave	55 (9.6%)	10 (10.2%)	0.994
S1Q3 pattern	34 (6.0%)	14 (14.3%)	0.006
QRS > 120 ms	51 (8.9%)	23 (23.5%)	< 0.001
QRS duration, ms	94 ± 18	102 ± 25	< 0.001
Complete right bundle branch block	31 (5.4%)	12 (12.2%)	0.020
Incomplete right bundle branch block	53 (9.3%)	7 (7.1%)	0.622
Left bundle branch block	17 (3.0%)	10 (10.2%)	0.002
Abnormal QRS axis	125 (21.9%)	46 (46.9%)	< 0.001
QRS axis, degrees	16 ± 48	−6 ± 52	< 0.001
Left anterior fascicular block	114 (20.0%)	39 (39.8%)	< 0.001
QRS fragmentation	5 (0.9%)	2 (2.0%)	0.61
Right ventricular hypertrophy	3 (0.5%)	1 (1.0%)	0.903
Left ventricular hypertrophy	7 (1.2%)	2 (2.0%)	0.863
Negative T waves	74 (13.0%)	19 (19.4%)	0.123
Non-specific repolarization abnormalities	82 (14.4%)	30 (30.6%)	< 0.001
Prolonged QTc	98 (17.2%)	29 (29.6%)	0.006
QTc duration, ms	435 ± 29	446 ± 36	0.006
Premature atrial complexes	43 (7.5%)	14 (14.3%)	0.044
Premature ventricular complexes	27 (4.7%)	4 (4.1%)	0.983

ECG, electrocardiogram; QTc, corrected QT interval. All results are presented as mean (%) or mean ± SD.

Supplementary Table 1 provides univariate analysis for identifying variables at admission associated with death. In multivariate analysis (Table 3), the presence of left bundle branch block remained statistically related to death [OR = 3.82, 95% confidence interval (CI): 1.52–9.28, *p* < 0.01], as did S1Q3 pattern (OR = 3.17, 95% CI: 1.38–7.03, *p* < 0.01) and repolarization abnormalities (OR = 2.41, 95% CI: 1.40–4.14, *p* < 0.01).

**Table 3:** Multivariate analysis for identifying variables at admission independently associated with death.

**Variables**	**OR**	**95% CI**	***p*-value**
**Model 1**			
Age (per 10 years)	1.98	1.62–2.46	< 0.001
Oxygen saturation (< 92 vs. ≥92%)	0.54	0.32–0.90	0.01
Dyspnea	3.25	1.92–5.66	< 0.001
Active cancer	5.94	2.73–12.96	< 0.001
Abnormal ECG at admission	2.28	1.15–4.92	0.02
**Model 2**			
Age (per 10 years)	1.81	1.46–2.28	< 0.001
Oxygen saturation (< 92 vs. ≥92%)	0.47	0.28–0.81	< 0.01
ECG at admission			
S1Q3 pattern	3.17	1.38–7.03	< 0.01
Left bundle branch block	3.82	1.52–9.28	< 0.01
Repolarization abnormalities	2.41	1.40–4.14	< 0.01

ECG, electrocardiogram; CI, confidence interval.

Model 1 was adjusted for age (per 10 years), oxygen saturation at admission, dyspnea, active cancer, and abnormal ECG at admission.

Model 2 was adjusted for age (per 10 years), oxygen saturation at admission, S1Q3 pattern at admission, left bundle branch block, and repolarization abnormalities at admission.

### Characteristics of ECG performed after admission

The characteristics of ECG performed after admission in 169 patients are presented in Table 4. An abnormal ECG was observed in 135 patients (79.9%) and 72 (42.9%) had a modified ECG as compared to the ECG at admission. The most frequent new ECG abnormality was T wave inversion (20 patients, 11.8%). Thirteen patients (7.7%) had a new supraventricular arrhythmia. Four patients (1.8%) presented a new S1Q3 pattern. Among the 169 patients who had a new ECG during hospitalization, 13 had a combination of hydroxychloroquine (HCQ) and azithromycin (AZT) regimen. The mean QTc interval was 443 ± 24 in patients under HCQ/AZT combination regimen vs. 437 ± 32 in patients without such treatment (*p* = 0.29). No patient in the HCQ/AZT group had a new prolonged QTC nor a QTc >500 ms. In multivariate analysis (Table 5), the occurrence of the following ECG parameters was associated with death: new repolarization abnormality (OR = 2.72, 95% CI: 1.14–6.54, *p* = 0.02), new S1Q3 pattern (OR = 13.23, 95% CI: 1.49–286.56, *p* = 0.03) and new-onset supraventricular arrhythmia (OR = 3.8, 95% CI: 1.11–13.35, *p* = 0.03).

**Table 4:** New ECG abnormalities observed during hospitalization, *n* = 169.

	**Alive,** ***n* = 135 (79.9%)**	**Dead,** ***n* = 34 (20.1%)**	***P* value**
Abnormal ECG	101 (74.8%)	34 (100%)	0.002
Modified ECG	50 (37.3%)	22 (64.7%)	0.007
New supraventricular arrhythmia	6 (4.4%)	7 (20.6%)	0.005
New atrioventricular block	2 (1.5%)	0 (0%)	1.000
New low voltage	2 (1.5%)	1 (2.9%)	0.493
New Q wave	2 (1.5%)	2 (5.9%)	0.181
New S1Q3 pattern	1 (0.7%)	3 (8.8%)	0.026
New QRS > 120 ms	1 (0.7%)	2 (5.9%)	0.103
New right bundle branch block	0 (0%)	2 (5.9%)	0.040
New incomplete right bundle branch block	2 (1.5%)	1 (2.9%)	0.493
New left bundle branch block	0 (0%)	0 (0%)	NA
New non-specific bundle block	0 (0%)	0 (0%)	NA
New abnormal QRS axis	5 (3.7%)	2 (5.9%)	0.629
New left anterior fascicular block	3 (2.2%)	2 (5.9%)	0.264
New right ventricular hypertrophy	1 (0.7%)	0 (0%)	1.000
New left ventricular hypertrophy	0 (0%)	0 (0%)	NA
New negative T wave	9 (6.7%)	11 (32.4%)	< 0.001
New non-specific repolarization abnormalities	10 (7.4%)	1 (2.9%)	0.696
New prolonged QTc	13 (9.6%)	6 (17.6%)	0.308
New premature atrial complexes	5 (3.7%)	4 (11.8%)	0.082
New premature ventricular complexes	3 (2.2%)	3 (8.8%)	0.097

ECG, electrocardiogram; QTc, corrected QT interval. All results are presented as value (%).

**Table 5:** Summary of multivariate analysis for identifying variables independently associated with death among patients who had a second ECG during hospitalization, *n* = 169.

**Variables**	**OR**	**95% CI**	***P* value**
**Model A**			
Age (per 10 years)	1.76	1.26–2.55	0.002
Modified ECG (as compared to baseline)	3.56	1.60–8.34	0.002
**Model B**			
Age (per 10 years)	1.81	1.41–2.29	< 0.001
New negative T wave	2.72	1.14–6.54	0.02
New S1Q3 pattern	13.23	1.49–286.56	0.03
New supraventricular arrhythmia	3.80	1.11–13.35	0.03

ECG, electrocardiogram; CI, confidence interval.

Model 1 was adjusted for age (per 10 years), and modified ECG.

Model 2 was adjusted for age (per 10 years), new negative T wave, new S1Q3 pattern, and new supraventricular arrhythmia.

## Discussion

In the present study, we evaluated the ECG characteristics of patients hospitalized with COVID-19. The main results are: (1) in a large cohort of COVID-19 patients, an abnormal ECG was observed in 478 patients (71.4%) and was more frequently described in patients who did not survive; (2) left bundle branch block, S1Q3 pattern and repolarization abnormalities at admission were associated with death; (3) new repolarization abnormality, new S1Q3 pattern and new-onset supraventricular arrhythmia during hospitalization were associated with death.

It is now well-known that ECG is modified by systemic inflammation, which may be consecutive to COVID infection or any of its complications (6, 8). In previous studies, an abnormal ECG was observed in between 47 and 93% of COVID-19 patients (23, 24, 26–28). In our study, 71.4% of patients presented with ECG abnormalities: 68.7% in patients admitted in medical wards and 79.3% in ICU patients. To our knowledge, no study reported on the proportion of ECG abnormalities in non-ICU COVID-19 patients. Even when these last patients presented with less severe COVID-19 involvement, most of them had ECG abnormalities and physicians should be aware that ECG should be systematically performed for a prognostic assessment. The observed differences between studies may be explained by different study populations, with a high prevalence of ECG abnormalities in the ICU. About one quarter of patients were admitted in ICU in the present study, which included all consecutive patients hospitalized in our university hospital during 2020.

In our study, CRP was not associated with ECG abnormality (*p* = 0.96), but was associated with new-onset supraventricular arrhythmia [mean CRP level was 163.4 mg/L in the group of patients with new-onset supraventricular arrhythmia vs. 107.2 mg/L in the group of patients without new-onset supraventricular arrhythmia (*p* = 0.04)]. This is consistent with previous studies, which identified systemic inflammation caused by COVID-19 as a key factor in arrhythmogenesis (14, 30). In the present study, new-onset supraventricular arrhythmia was associated with death in multivariate analysis which is consistent with previous studies (24, 26, 31). In our study, no significant difference in QTc duration was found between patients with HCQ/AZT combination therapy and patients without treatment. This is in contradiction with the study by Bernardini et al. (32); where the HCQ/AZT combination therapy caused a significantly increase of QT interval compared to HCQ alone or no treatment group. This may be due to the fact that in our institution, we prescribed HCQ/AZT combination regimen during a limited period (04/06/2020 to 04/16/2020) in a limited number of patients (n = 13) and ECG was not systematically performed. Other ECG abnormalities that we describe in our study are consistent with the literature (26–28, 33). Interestingly, we found that left bundle branch block, S1Q3 pattern and repolarization abnormalities were independently associated with death in cases of COVID-19.

ECG abnormalities may be associated with death by many mechanisms. Systemic inflammation can increase cardiac demand, and destabilize vascular plaque, resulting in myocardial infarction (17–20), and thereby cause T wave inversion or non-specific repolarization abnormalities, which can worsen the prognosis (8). In a postmortem study (21), the most common pathological cause of myocyte necrosis in patients with COVID-19 infection was microthrombi. Indeed, only 3 patients in this study had a ST-elevation myocardial infarction, supporting the hypothesis of intra-coronary microthrombi rather than macrothrombus. Another mechanism of death during COVID-19 is pulmonary embolism. It is now well-established that COVID-19 significantly increases the risk of pulmonary embolism (11, 34). In our study, S1Q3 pattern was statistically associated with death. This S1Q3 pattern is not pathognomonic for pulmonary embolism, but the occurrence of this ECG parameter may be helpful for the risk stratification of COVID-19 patients.

Potential limitations of the present study merit consideration. First, we recruited patients presenting with COVID-19 from the first wave and we did not study the ECG characteristics associated with new variants. Even if the prognosis of COVID-19 caused by these new variants is better as compared to the first wave (4), the most recent waves were accompanied by great contagiousness and many hospitalizations (3, 4). Second, in our study, ECG was not systematically repeated during hospitalization, and we analyzed at least two ECG during hospitalization in 169 selected patients, leading to a potential bias of interpretation. However, experts blinded to clinical data and outcome interpreted the ECG, and the occurrence of new ECG abnormalities was associated with death and was consistent with the results of ECG at admission. Finally, the low number of patients presenting with new S1Q3 pattern during hospitalization does not allow to definitively conclude on the prognostic impact of S1Q3 pattern.

## Conclusion

The presence of abnormal ECG during COVID-19 is frequent. Several ECG abnormalities such as left bundle branch block, S1Q3 pattern, repolarization abnormalities and supraventricular arrhythmia are associated with death as well as the occurrence of ECG abnormalities during hospitalization. Clinicians should be aware of the usefulness of ECG for risk stratification during COVID-19.

## Data availability statement

The raw data supporting the conclusions of this article will be made available by the authors, without undue reservation.

## Ethics statement

The studies involving human participants were reviewed and approved by University Paris Saclay Institutional Review Board (CER-Paris-Saclay-2021-102). The patients/participants provided their written informed consent to participate in this study.

## Author contributions

MH-M, GC, and NM contributed to conception and design of the study. GC and MH-M analyzed ECG and wrote the first draft of the manuscript. SG, AC, VA, A-SL, and MO obtained data. GC organized the database. MH-M, NM, and HH performed the statistical analysis. NM, SB, and OD wrote sections of the manuscript. All authors contributed to manuscript revision, read, and approved the submitted version.

## Conflict of interest

The authors declare that the research was conducted in the absence of any commercial or financial relationships that could be construed as a potential conflict of interest.

## Publisher's note

All claims expressed in this article are solely those of the authors and do not necessarily represent those of their affiliated organizations, or those of the publisher, the editors and the reviewers. Any product that may be evaluated in this article, or claim that may be made by its manufacturer, is not guaranteed or endorsed by the publisher.
