# Supplementary material for: Electrocardiogram abnormalities and prognosis in COVID-19
Source: Front Cardiovasc Med. 2022 Oct 5;9:993479. doi: 10.3389/fcvm.2022.993479 (PMC9581294; doi:10.3389/fcvm.2022.993479)
Supplement: Supplementary file 1 [file Table_1.docx]

**Supplementary Table 1.** Univariate analysis for identifying variables at admission associated with death

| **Variables** | **OR** | **95% CI** | **p-value** |
| --- | --- | --- | --- |
| Age (per 10 years) | 1.93 | 1.61-2.33 | <0.001 |
| Hypertension | 2.57 | 1.65-4.08 | <0.001 |
| Coronary artery disease | 2.11 | 1.19-3.64 | 0.0086 |
| Heart failure | 4.33 | 1.90-9.56 | <0.001 |
| Active cancer | 4.72 | 2.40-9.11 | <0.001 |
| Dyspnea | 2.53 | 1.59-4.14 | <0.001 |
| Oxygen saturation (<92 vs ≥92%) | 0.40 | 0.26-0.63 | <0.001 |
| ECG at admission |  |  |  |
| Abnormal ECG | 3.69 | 2.01-7.47 | <0.001 |
| Supraventricular arrhythmia | 1.64 | 0.84-3.01 | 0.127 |
| Atrioventricular block, first degree | 2.67 | 1.39-4.92 | 0.002 |
| Complete right bundle branch block | 2.40 | 1.15-4.74 | 0.015 |
| Left bundle branch block | 3.65 | 1.57-8.11 | 0.0018 |
| S1Q3 pattern | 2.60 | 1.30-4.95 | 0.0048 |
| Repolarization abnormalities | 2.53 | 1.62-3.92 | <0.001 |
| Prolonged QTc | 2.00 | 1.22-3.21 | 0.0052 |
| Premature atrial complexes | 2.02 | 1.03-3.76 | 0.033 |

ECG: electrocardiogram; CI: confidence interval; QTc: corrected QT interval
